# Evaluation Effect of Aspiration of 0.2 ml of Cerebrospinal Fluid After Completion of Injection 0.5% Bupivacaine and Reinjection Into Subarachnoid Space on Sensory and Motor Block in Cesarean Section: A Randomized Clinical Trial

**DOI:** 10.3389/fmed.2022.816974

**Published:** 2022-03-25

**Authors:** Nahid Manouchehrian, Zahra Miri, Farzaneh Esna-Ashari, Farshid Rahimi-Bashar

**Affiliations:** ^1^Anesthesia and Critical Care Department, Hamadan University of Medical Sciences, Hamadan, Iran; ^2^Department of Family and Community Medicine, Faculty of Medicine, Hamadan University of Medical Sciences, Hamadan, Iran

**Keywords:** cerebrospinal fluid, spinal anesthesia, sensory block, motor block, cesarean section

## Abstract

**Background:**

Spinal anesthesia (SPA) is the most common type of anesthesia administered for cesarean section. The main aim of this study was to evaluate the effect of aspiration of CSF (0.2 mL) immediately after SPA with hyperbaric 0.5% bupivacaine on the extent of sensory and motor block.

**Methods:**

In this clinical trial, 60 women at ≥37 weeks of gestation and aged between 18 and 46 years, candidate for cesarean delivery under spinal anesthesia were randomly allocated into two equal groups (*n* = 30). Group A (CSF-aspiration group) received the spinal anesthesia with 10 mg of hyperbaric 0.5% bupivacaine with aspiration of 0.2 ml of CSF. Group B (no-CSF-aspiration group) received only 10 mg of 0.5% hyperbaric bupivacaine. Pin-prick analgesia and motor block were tested during the induction.

**Results:**

The mean maximum level of analgesia was T6 in each group. Although the mean time to reach the maximum level of anesthesia (4.43 ± 5.14 vs. 2.76 ± 2.04, *P* = 0.107) and to reach T10 level (50.56 ± 11.51 vs. 49.10 ± 13.68, *P* = 0.665) in the CSF-aspiration group is longer than the non-CSF-aspiration group, but this differences were not significant. There were no significant between-group differences regarding sensory and motor block quality (*P* = 0.389) or failed SPA (four cases in CSF-aspiration group vs. two cases in no-CSF-aspiration group, *P* = 0.389). The incidence of bradycardia, hypotension, headache, vomiting and nausea were similar in both groups (*P* > 0.05). In addition, the difference in hemodynamic parameters between the two groups over times was not statistically significant.

**Conclusion:**

Our finding indicated that the aspiration of 0.2 ml of CSF after injection of spinal anesthesia with hyperbaric 0.5% bupivacaine does not seem to affect the extent of sensory and motor block, success rate, or outcome after SPA in cesarean section.

**Clinical Trial Registration:**

[https://www.irct.ir/search/result?query=IRCT20120915010841N25], identifier [IRCT20120915010841N25].

## Introduction

Cesarean delivery (C-section) is one of the methods to termination of pregnancy that is performed under general or regional anesthesia (1). General anesthesia has become less commonly used in recent decades due to the widespread utilization of regional anesthesia methods and the understanding that these techniques can be provided even in an emergency situation (2). Despite recent devices that facilitate endotracheal intubation and clinical algorithms, guiding anesthesiologists still facing significant challenging scenarios, risks, and complications of general anesthesia for both mother and neonate(s) at the time of delivery (3).

Today, the pervasive method in cesarean section is regional anesthesia, which is usually a safer option than general anesthesia (4). Spinal anesthesia (SPA), epidural anesthesia, and combined spinal-epidural anesthesia (CSE) are the three regional anesthetic techniques available for cesarean delivery (5, 6). Regional anesthesia especially SPA has been favored as the best choice for elective cesarean delivery due to its faster onset of action, simpler technique, more complete sensory and motor block, greater maternal comfort, infant safety, and less risk of aspiration of gastric content (7, 8). This method is safe and effective, but it is not a 100% successful method. Hypotension (9), post-dural puncture headache (PDPH) (10), bradycardia (11), nerve damage (12), nausea and vomiting (13), high level of anesthesia and failure in SPA are the most common complications (14).

Failed spinal anesthesia can be defined as partial or incomplete spinal block within 15–20 min after injection and requiring supplemental analgesia or conversion to general anesthesia (15, 16). Evidence showed that the incidence of failure SPA is between 1 and 17% (17, 18). History of previous anesthesia, obesity, dry tap of cerebrospinal fluid (CSF), bloody CSF, miscalculation of the dose, maldistribution of the local anesthetic, multiple lumbar puncture attempts, use of the L4/L5 interspace and technical errors were shown to significantly associate with failed spinal anesthesia (14, 19, 20).

The control of anesthetic level with both hyperbaric and isobaric bupivacaine that used for spinal block is always difficult. In previous studies, the effect of different volumes, dosages, and concentrations on the spread of sensory and motor blocks has been investigated and has shown wide variability (21–24). Among these factors, the effect of CSF aspiration injection on the distribution of local anesthesia remains controversial and there is little information about its effectiveness in quantitative terms (25–27). Therefore, we conducted this study to evaluate the effect of aspiration of 0.2 mL of CSF immediately after injection of 2.5 ml hyperbaric 0.5% bupivacaine in subarachnoid space on the extent of sensory and motor block level in cesarean section under spinal anesthesia.

## Materials and Methods

### Study Design

This prospective, randomized, double blind, controlled clinical trial study was conducted in Fatemiyeh Hospital in Hamadan, Iran, between 2019 and 2020. The protocol study was reviewed and approved by the Ethics Committees of Hamadan University of Medical Sciences (IR.UMSHA.REC.1399.630). This study Registered at Iranian Registry of Clinical Trials (IRCT20120915010841N25). Written informed consent were obtained from each patient. The study was conducted in accordance with the Declaration of Helsinki and subsequent (28).

### Participants

American Society of Anesthesiologists (ASA) physical status I–II term nulliparous women at ≥37 weeks of gestation with a singleton pregnancy and aged between 18 and 46 years, who presented for cesarean delivery under spinal anesthesia were included in this study. Any patients not willing to participate in this study, presence of any contraindication for SPA including with spinal deformities, inability to maintain the required body position during needle puncture, elevated intracranial pressure, localized infection at the site of needle insertion, severe allergies to local anesthetics, neurological disorders, and acute comorbidities such as severe hypotension (mean arterial pressure <50 mm/Hg), cardiovascular diseases (ejection fraction <30%), liver diseases (liver enzyme levels 1.5 times higher than normal levels), and renal diseases (creatine >1.5 mg/dl) were excluded from the study.

### Sample Size

The sample size of this study was calculated based on previous studies was conducted by Bjurström et al. (27), showed that the reduction of CSF volume by 10 mL increased the extent of sensory anesthesia (mean thoracic level 4.3 ± 2.4 vs. 7.1 ± 2.6, *P* < 0.001). We used formula for difference in proportions between two groups, and concerning the Type I error (α) set as two-sided 5% (Z1–α/2 = 1.96), type II error (β) set as 20% (Z1–β = 0.84) and power of 80%. A sample size of 60 patients, 30 in each arm, is sufficient to detect a clinically important difference between two groups.


(1) $$n=\frac{\left(sd1^{2}+sd2^{2}\right)+{\left(Z1{-}_{\alpha }/2+\text{Z}1{-}_{\beta }\right)}^{2}}{{\left(\mu 1-\mu 2\right)}^{2}}$$

μ1: Mean of thoracic level before intervention (4.3).
μ2: Mean of thoracic level after intervention (7.1).
Sd1: standard deviation of the thoracic level before intervention (2.4).
Sd2: standard deviation of the thoracic level after intervention (2.6).

### Randomization and Blinding

Women at more than 37 weeks of gestation with a singleton pregnancy who met the all criteria for inclusion were randomly assigned to the Group A or B based on block randomization. Group A (CSF-aspiration group) underwent spinal anesthesia with hyperbaric 0.5% bupivacaine and immediately afterward received aspiration of 0.2 mL of CSF. Group B (no-CSF-aspiration group) received only spinal anesthesia with hyperbaric 0.5% bupivacaine. Block randomization was performed using sealed envelope technique and computer generated random numbers by Random Allocation Software (RAS; Informer Technologies, Inc., Madrid, Spain). In addition, the researchers and nurses who completed the data collection forms were blinded to the group allocated of patients.

### Anesthesia Procedure and Data Collection

Before the administration of anesthesia drug, ringer serum (10 ml/kg) was injected into a suitable peripheral cubital vein. In sitting position, spinal puncture was applied in lumber (L3-L4 or L4-L5 region) including 2.5 ml hyperbaric 0.5% bupivacaine using a 25-G Quincke pencil point spinal needle. In group A (CSF-aspiration group), at the end of the anesthesia drug injection, 0.2 mL of CSF was re-aspirated and re-injected into the subarachnoid space. In group B (no-CSF-aspiration group), after the injection of the anesthesia drug without CSF aspiration, the spinal needle was removed immediately after the injection and the patients were turned to the supine position.

Variables assessed included demographic such as age and clinical variables included cesarean reasons, gravida, hemodynamic parameters, duration of surgery, spinal anesthesia time, time to reach maximum level of anesthesia, time to reach the tenth thoracic vertebra (T10) level, sensory and motor block quality, maximum level sensory block according to thoracic vertebra, high spinal, failed SPA, sensory level in recovery and SPA-related complications (such as hypotension, PDPH, bradycardia, nausea, and vomiting) were recorded for each patients. All parameters were noted by an anesthesiologist and nurses blinded to the group allocation.

Standard monitoring including electrocardiography, pulse oximetry and non-invasive blood pressure (NIBP) monitoring was performed for each patient. Systolic blood pressure (SBP), diastolic blood pressure (DBP), mean arterial pressure (MAP), and heart rates (HR) were measured and recorded using an X162 monitor (Saadat Co., Tehran, Iran) at baseline (Time 0) and immediately after spinal anesthesia (T1) and then every 2 min and up to 10-min (T2–T6), and then every 5 min up to 30-min (T7–T10), and then every 10 min up to 60-min (T11–T13) min after infusion of an anesthesia drug. So that each of the hemodynamic parameters from the time before the injection of anesthesia to 1 h after the injection of anesthesia was recorded 14 times for each patient. Any fall in the SBP below 90 mmHg or a fall in MAP of more than 20% from baseline value was taken as hypotension. Hypotension and bradycardia (heart rate less than 60 beats per minute), managed with intravenous 10, 20, or 30 mg of ephedrine and 0.5 or 1 mg atropine, respectively. The amount of ephedrine and atropine used were also recorded.

Maximum level of sensory block, was evaluated by a pin-prick method using a 25-gauge needle and time to reach maximum level of anesthesia and time to reach T10 level was recorded for each patients. Quality of sensory block was evaluated by the visual analog scale (VAS); scores were recorded by making a handwritten mark on a 10-cm line that represents a continuum between “excellent” and “poor” (29). VAS scores described postoperative excellent quality as none (0), mild (<3), moderate (3–6), or poor (7–10). Quality of motor block was evaluated by the Bromage Scale (30). The modified Bromage Scale was used: 0 = no motor block; 1 = able to flex knee free movement of feet, unable to raise extended leg (partial motor block); 2 = free movement of feet only (almost complete motor block); 3 = unable to move hips, knees, feet (complete motor block).

Sedation was assessed by Ramsay scale, it divides a patient’s level of sedation into six categories ranging from severe agitation to deep coma; 1 = anxious and agitated or restless or both; 2 = co-operative, oriented and tranquil; 3 = responding to commands only; 4 = brisk response to light glabellar tap or loud auditory stimulus; 5 = sluggish response to light glabellar tap or loud auditory stimulus; 6 = No response to stimulus (31).

If, within 20 min of the initial spinal anesthesia, no signs of sensory or motor block are observed and confirmed by the anesthesiologist to initiate surgery, the patient will be a candidate for second SPA or general anesthesia (GA) and will be registered as a spinal failure in the questionnaire. Also, in cases where the level of anesthesia is more than the required amount for cesarean section (above T4) supportive measures are taken for the mother and she was recorded in the questionnaire as high spinal. In addition, neonatal Apgar score in minutes 1 and 5 were checked and recorded.

### Statistical Analysis

The Shapiro–Wilk test was conducted to test whether the data were normally distributed. Descriptive baseline characteristics for two group comparisons were tabulated as the mean ± standard deviation (SD) or as percentages. A chi-square or Fisher’s exact test was performed for comparisons between two groups of categorical data. Continuous data were statistically analyzed using a *t*-test or Mann-Whitney *U* test. Primary efficacy data were examined using an intention-to-treat analysis. Using a general linear model, hemodynamic changes and complications between the two groups were compared using a repeated measurement ANOVA test, with the baseline values (age, cause of cesarean, and gravida) used as covariates in the model. The assumption of sphericity was addressed by Mauchly’s test of sphericity, and when the assumption was not satisfied, the Greenhouse–Geisser correction of *P*-value were utilized. To assess the effect of intervention, the analysis of covariance (ANCOVA) was used after controlling for baseline measures and confounders in a two-step hierarchical model. For the ordinal primary outcome, the ordinal regressions were utilized after controlling for baseline measures and confounders in a two-step hierarchical model. Statistical analysis was carried out using SPSS software (ver. 21) (SPSS Inc., Chicago, IL, United States). In all analyses, *P*-values less than 0.05 were considered as significant.

## Results

### Participants of Study

**FIGURE 1:**
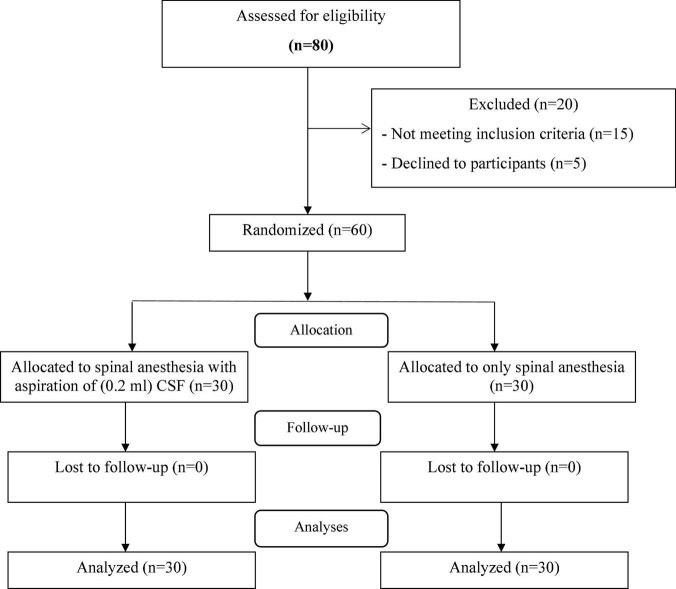
CONSORT flow diagram.

A total of sixty parturient women were enrolled in the study. The enrollment flow chart of patients is presented in Figure 1. Eighty physical status I–II ASA term nulliparous women at ≥37 weeks of gestation with a singleton pregnancy and aged between 18 and 46 years, in Fatemiyeh Hospital in Hamadan, Iran, screened for eligibility criteria. Out of 80 cases, 60 patients met the inclusion criteria and randomly assigned into two groups with 30 patients in each group; CSF-aspiration group (received spinal anesthesia with 10 mg of hyperbaric 0.5% bupivacaine with aspiration of 0.2 ml of CSF immediately afterward) and no-CSF-aspiration group (received only spinal anesthesia with 10 mg of hyperbaric 0.5% bupivacaine). During the intervention and follow-up stages, no patient was excluded from the study and finally 30 patients in each group were analyzed.

### Baseline Demographic and Clinical Characteristics

Table 1 shows the baseline demographic and clinical characteristics of the study participants in two groups of study. There were no statistically significant differences in baseline demographic and clinical characteristics of parturient such as; age (*P* = 0.919), cause of cesarean (*P* = 0.201) and gravida (*P* = 0.055). In terms of hemodynamic parameters, statistical significant differences was observed between HR (*P* < 0.001) and systolic BP (*P* = 0.01) in the CSF-aspiration and no-CSF-aspiration groups.

**TABLE 1:** Baseline demographic and clinical characteristics in two groups of study.

Variables		CSF-aspiration group (*n* = 30)	No-CSF-aspiration (*n* = 30)	*P*-value	95% confidence interval (CI)
Age (Years)	Mean ± SD	31.40 ± 8.73	31.60 ± 6.16	0.919	−4.10 to 3.70
	(Range)	(18–64)	(19–44)		
Cesarean causes	Repeated	20 (66.7)	23 (76.7)	0.201	-
	High risk	7 (23.3)	7 (23.3)		
	Infertility	3 (10)	0		
Gravida	Mean ± SD	2.83 ± 1.26	2.30 ± 0.79	0.055	−0.011 to 1.078
	(Range)	(1–5)	(1–4)		
Hemodynamic parameters	HR	104.30 ± 15.17	90.40 ± 15.95	<0.001*	5.85 to 21.94
	Systolic BP	129.10 ± 13.08	121.56 ± 8.43	0.010*	1.84 to 13.22
	Diastolic BP	79.20 ± 13.16	77.33 ± 9.83	0.581	−4.34 to 7.67
	MAP	95.36 ± 12.26	91.20 ± 9.15	0.141	−1.42 to 9.75

*Statistically significant. HR, heart rate; BP, blood pressure; MAP, mean arterial pressure.

### Spinal Anesthesia Characteristics and Outcomes

Comparison of spinal anesthesia characteristics and outcomes in two groups of study are presented in Table 2. According to the results, there were no significant differences between the two groups with respect to sensory and motor block quality (*P* = 0.398). Excellent and complete quality of sensory and motor blocks was observed in more than 80% of patients, respectively.

**TABLE 2:** Comparison of spinal anesthesia characteristics and outcomes in two groups of study.

Variables		CSF-aspiration group (*n* = 30)	No-CSF-aspiration group (*n* = 30)	*P*-value
Operation time	Mean ± SD (min)	54.96 ± 13.17	51.86 ± 13.85	0.378
Time to reach maximum level of anesthesia	Mean ± SD (min)	4.43 ± 5.14	2.76 ± 2.04	0.107
Time to reach T10 level	Mean ± SD (min)	50.56 ± 11.51	49.10 ± 13.68	0.665
Maximum sensory level	T4	3 (10.0)	1 (3.3)	0.612
	T6	26 (86.7)	29 (96.7)	
	T8	1 (3.3)	0	
Sensory block quality	Excellent (%)	26 (86.7)	28 (93.3)	0.389
	Moderate (%)	1 (3.3)	0	
	Poor (%)	3 (10.0)	2 (6.7)	
Motor block quality	Complete (%)	26 (86.7)	28 (93.3)	0.389
	Semi-complete (%)	1 (3.3)	0	
	Non-motion block (%)	3 (10.0)	2 (6.7)	
Ephedrine consumed (%)	10 mg	6 (20)	7 (23.3)	
	20 mg	8 (26.7)	6 (20)	0.849
	30 mg	1 (3.3)	1 (3.3)	
Atropine consumed (%)	0.5 mg	2 (6.7)	0	0.248
	1 mg	1 (3.3)	1 (3.3)	
Fail spinal	Yes (%)	4 (13.3)	2 (6.7)	0.389
Apgar score	Mean ± SD (1 min)	8.86 ± 0.345	8.93 ± 0.25	0.398
	Mean ± SD (5 min)	9.86 ± 0.345	9.93 ± 0.25	0.398
Ramsay scale	Mean ± SD	2.10 ± 0.661	2.16 ± 0.53	0.668

*T4, fourth thoracic vertebra; T6, sixth thoracic vertebra.*

The maximal level of anesthesia was almost identical T6 in each group. Though the mean ± SD (min) time taken to reach the maximal anesthesia level (4.43 ± 5.14 vs. 2.76 ± 2.04) and also time to reach T10 level (50.56 ± 11.51 vs. 49.10 ± 13.68) were obviously longer in the CSF-aspiration group than in the no-CSF-aspiration group. However, this differences were not reach to statistically significant, (*P* = 0.107 and *P* = 0.665). In addition, according to ANCOVA adjusted for age, cesarean reasons and gravida, time to reach the maximum anesthesia level and also time to reach T10 level was not significant difference in two group of study (Table 3).

**TABLE 3:** Univariate effect of collected variables on time to reach maximum level of anesthesia and time reach T10 level.

	Variables	SS	df	MS	*F*	*P*-value	η^2^
Time to reach maximum level of anesthesia	Group (intervention vs. control)	32.30	1	32.30	1.974	0.166	0.034
	Cesarean reasons	13.80	2	6.903	0.422	0.658	0.016
	Age	3.085	1	3.085	0.189	0.666	0.004
	Gravida	0.194	1	0.194	0.012	0.914	000
Time to reach T10 level	Group (intervention vs. control)	1.423	1	1.423	0.009	0.923	000
	Cesarean reasons	377.23	2	188.619	1.238	0.298	0.945
	Age	156.39	1	156.39	1.027	0.315	0.019
	Gravida	567.82	1	567.82	3.728	0.059	0.066

*SS, sum of square; df, degrees of freedom; MS, mean square; η^2^, partial eta square.*

Four patients (13.3%) in the CSF-aspiration group (intervention group) and two patients (6.7%) in the no-CSF-aspiration group experienced failed SPA. There was no significant difference between groups regarding incidence of failed SPA (*P* = 0.389). According to Table 2, the mean ± SD of Apgar showed no significant difference between the two groups in the first minute (8.86 ± 0.345 vs. 8.93 ± 0.25) and 5 min (9.86 ± 0.345 vs. 9.93 ± 0.25) based on the *t*-test (*P* = 0.398). In addition, no significant difference was observed between the two groups in terms of using ephedrine and atropine (*P* < 0.05).

### Changes in Hemodynamic Parameters Over Time

**FIGURE 2:**
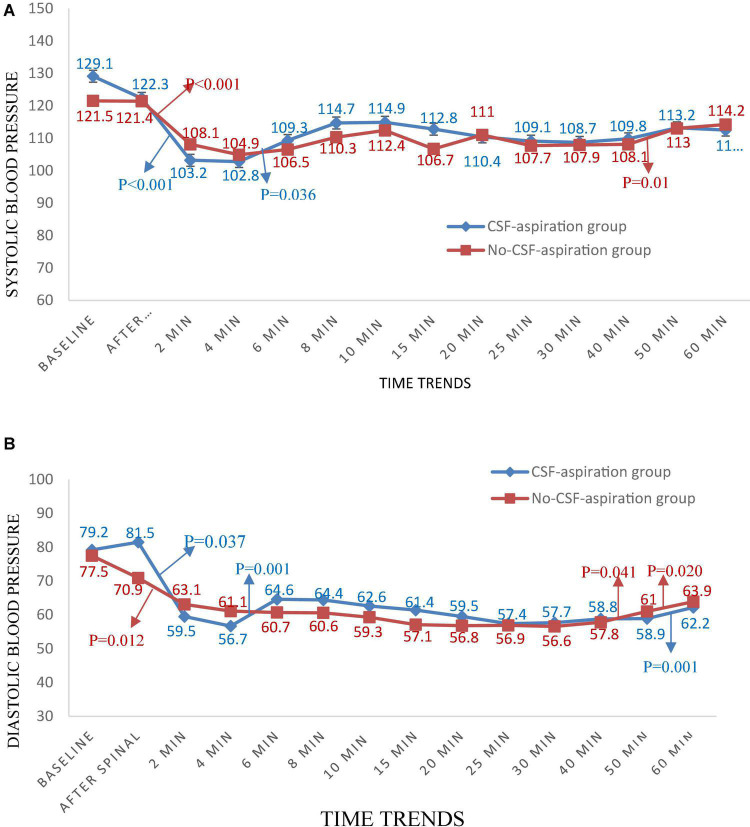
Changes **(A)** systolic and **(B)** diastolic blood pressure in two groups of study over times, *P-values shows statistically significant between two times within groups.

**FIGURE 3:**
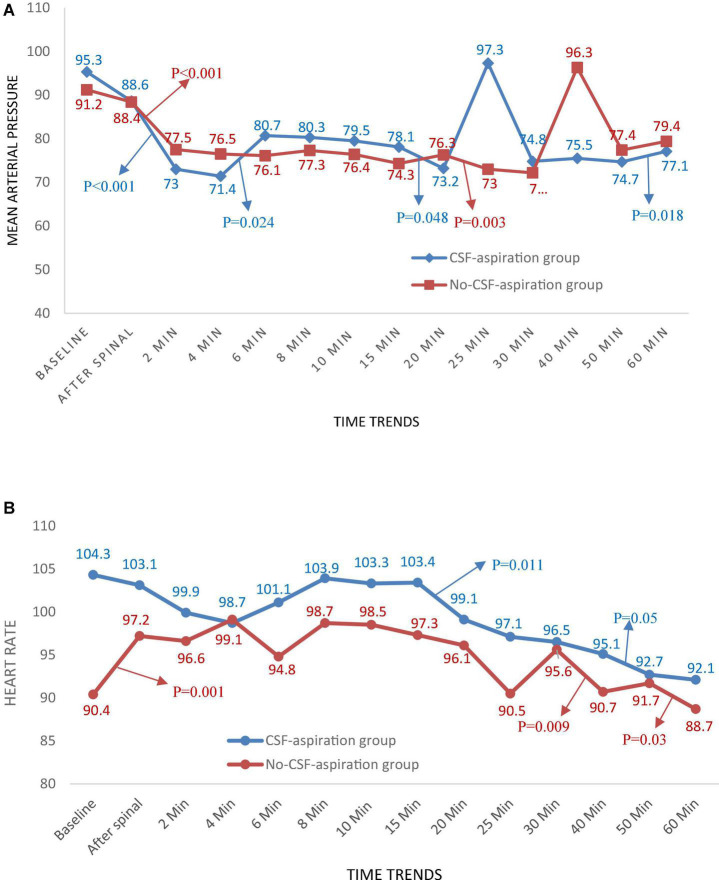
Changes **(A)** main arterial pressure and **(B)** heart rate in two groups of study over times, *P-values shows statistically significant between two times within groups.

Time trends of hemodynamic parameters in two groups of study are presented in Table 4. Hemodynamic parameters (systolic BP, diastolic BP, MAP, and HR) were recorded at baseline and immediately after spinal anesthesia and then every 2 min up to 10-min, and then every 5 min up to 30-min (T7-T10), and then every 10 min up to 60-min after infusion of an anesthesia drug. Figure 2A shows the mean values for changes of systolic BP in each group over times. At baseline the systolic BP was significantly higher in the CSF-aspiration group compared to the no-CSF-aspiration group (129.1 ± 13.1 vs. 121.5 ± 8.43, *P* < 0.01), while the difference was not statistically significant between other times (*P* > 0.05). In within group, time effect on SBP was statistically significant in each group (a within-subject difference or time effect) (*P* < 0.001). As shown in Figure 2B, there was a statistically significant time trend (a within-subject difference or time effect) for diastolic BP in each group (*P* < 0.001). However, the trend in changes in diastolic BP levels was not statistically significant between two groups (group × time interaction or an interaction effect) (*P* = 0.518). Figures 3A,B shows the mean values for changes of MAP and HR in each group over times, respectively. There was a statistically significant time trend (a within-subject difference or time effect) for MAP in both groups (*P* < 0.001). However, the trend in changes in MAP levels was not statistically significant between two groups (group × time interaction or an interaction effect) (*P* = 0.324). At baseline the HR was significantly higher in the CSF-aspiration group compared to the no-CSF-aspiration group (104.3 ± 15.1 vs. 90.4 ± 15.9, *P* = 0.01), while the difference was not statistically significant between other times (*P* > 0.05). In within group, time effect on HR was statistically significant only in the CSF-aspiration group (*P* < 0.002).

**TABLE 4:** Comparison of hemodynamic parameters in the intervention and control groups according to time trends.

Time trends	Systolic BP		*P-value	Diastolic BP		*P-value	MAP		*P-value	HR		*P-value
Mean (SD)	Group A	Group B		Group A	Group B		Group A	Group B		Group A	Group B	
Baseline	129.1 (13.1)	121.5 (8.43)	0.010^#^	79.2 (13.1)	77.5 (9.83)	0.581	95.3 (12.2)	91.2 (9.15)	0.141	104.3 (15.1)	90.4 (15.9)	0.001^#^
After spinal	122.3 (19.6)	121.4 (12.5)	0.833	81.5 (49.9)	70.9 (17.6)	0.279	88.6 (20.3)	88.4 (11.8)	0.957	103.1 (20.2)	97.2 (15.5)	0.217
After 2 min	103.2 (19.2)	108.1 (16.4)	0.297	59.5 (16.2)	63.1 (13.7)	0.362	73.1 (17.5)	77.5 (14.5)	0.287	99.9 (22.1)	96.6 (20.8)	0.558
After 4 min	102.8 (18.9)	104.9 (18.4)	0.665	56.7 (16.6)	61.1 (13.6)	0.272	71.4 (17.8)	76.5 (14.2)	0.229	98.7 (20.9)	99.1 (18.7)	0.954
After 6 min	109.3 (16.8)	106.5 (16.2)	0.519	64.6 (14.6)	60.7 (11.5)	0.260	80.7 (19.8)	76.1 (13.2)	0.295	101.1 (21.5)	94.8 (24.6)	0.294
After 8 min	114.7 (14.1)	110.3 (15.4)	0.254	64.4 (14.8)	60.6 (11.1)	0.263	80.3 (12.5)	77.2 (11.3)	0.380	103.9 (18.4)	98.7 (18.5)	0.278
After 10 min	114.9 (13.7)	112.4 (12.6)	0.465	62.6 (13.2)	59.3 (11.2)	0.301	79.5 (12.6)	76.4 (12.5)	0.334	103.3 (16.2)	98.5 (14.6)	0.230
After 15 min	112.8 (13.4)	106.7 (22.2)	0.200	61.4 (12.4)	57.1 (10.9)	0.154	78.1 (12.3)	74.3 (11.5)	0.233	103.4 (15.6)	97.3 (15.7)	0.143
After 20 min	110.4 (11.2)	111.0 (13.1)	0.867	59.5 (12.4)	56.8 (12.6)	0.402	73.2 (16.9)	76.3 (11.6)	0.416	99.1 (14.5)	96.1 (12.2)	0.396
After 25 min	109.1 (12.6)	107.7 (11.8)	0.652	57.4 (11.9)	56.9 (11.7)	0.888	97.3 (12.5)	73.0 (11.1)	0.308	97.1 (13.1)	90.5 (18.5)	0.118
After 30 min	108.7 (10.4)	107.9 (12.8)	0.809	57.7 (9.9)	56.6 (11.4)	0.711	74.8 (9.84)	72.2 (11.5)	0.351	96.5 (15.6)	95.6 (14.1)	0.816
After 40 min	109.8 (10.5)	108.1 (11.2)	0.541	58.8 (9.9)	57.8 (11.7)	0.706	75.5 (10.2)	96.3 (12.7)	0.352	95.1 (15.5)	90.7 (11.6)	0.225
After 50 min	113.2 (19.1)	113.0 (10.2)	0.960	58.9 (9.8)	61.0 (10.1)	0.419	74.7 (9.26)	77.4 (9.38)	0.273	92.7 (14.8)	91.7 (14.5)	0.793
After 60 min	112.5 (10.2)	114.2 (9.13)	0.491	62.2 (10.5)	63.9 (8.20)	0.472	77.1 (9.80)	79.4 (9.51)	0.362	92.1 (16.2)	88.7 (10.2)	0.354
*P*-value**	<0.001^#^	<0.001^#^		<0.001^#^	<0.001^#^		<0.001^#^	<0.001^#^		0.002^#^	0.494	
*P*-value ***	<0.001^#^			<0.001^#^			0.183			0.239		
*P*-value ****	0.249			0.518			0.324			0.363		

Group A, CSF-aspiration group; Group B, non-CSF-aspiration group; BP, blood pressure; MAP, mean arterial pressure; HR, heart rate. ^#^P < 0.05 was considered as significant. *P-value based on independent t-test and analysis of covariance (ANCOVA) adjusted for age, caesarian reasons and Gravida between two groups. **P-value based on paired t-test within group. ***Time main effect based on two way analysis of variance with repeated measures (RMANOVA). ****Assessing the interaction effect of group and time based on RMANOVA after Greenhouse–Geisser correction (adjusted and non-adjusted models).

### Spinal Anesthesia-Related Complications

Table 5 shows comparison of SPA-related complications during operation and recovery in two groups of study. According to our findings, hypotension is a common side effect of SPA and it occurred in 17 patients (56.7%) and 14 patients (46.7%) in the CSF-aspiration and non-CSF-aspiration groups, respectively. The frequency distribution of nausea and vomiting in both groups of study was similar (23.3%). There were no significant differences between the two groups with respect to SPA-related complications during operation and recovery (*P* > 0.05).

**TABLE 5:** Comparison of SPA-related complications during operation and recovery in two groups of study.

Side effects	CSF-aspiration group (*n* = 30)	No-CSF-aspiration group (*n* = 30)	*P*-value
**During operation**			
Nausea and vomiting	7 (23.3)	7 (23.3)	1.000
Headache	1 (3.3)	4 (13.3)	0.353
Hypotension	17 (56.7)	14 (46.7)	0.438
Bradycardia	4 (13.3)	2 (6.7)	0.671
**Recovery**			
Nausea and vomiting	0	1 (3.3)	0.355
Headache	1 (3.3)	1 (3.3)	1.000

### Exploratory Correlation Analyses

We found no significant relationships between groups (intervention vs. control), cause of cesarean (repeated vs. infertility or high risk vs. infertility), and sensory block quality or motor block quality. The results of ordinal regressions for sensory and movement block quality are shown in Table 6.

**TABLE 6:** The results of ordinal regressions for sensory and movement block quality.

	Variables	Parameter estimate	95% confidence interval (CI)		*P*-value
			Lower	Upper	
Sensory block quality	Groups (Intervention vs. control)	0.846	−0.939	2.632	0.353
	Cesarean reasons (Rep. vs. infertility)	−0.371	−2.231	1.489	0.696
	Cesarean reasons (High risk vs. infertility)	−18.85	−8.85	4.256	0.696
Movement block quality	Groups (Intervention vs. control)	0.846	−0.939	2.632	0.353
	Cesarean reasons (Rep. vs. infertility)	−0.371	−2.231	1.489	0.696
	Cesarean reasons (High risk vs. infertility)	−18.85	−8.85	4.256	0.696

## Discussion

The main objective of this clinical trial study was to evaluate the effect of CSF aspiration to SPA on extent of sensory and motor block. Our findings indicated that the aspiration of 0.2 ml of CSF after injection of spinal anesthesia with hyperbaric 0.5% bupivacaine had no effect on the extent of sensory and motor block, success rate, or outcome after SPA in cesarean section. The mean maximum level of analgesia was T6 in each group. The excellent quality of sensory block and the complete quality of motor block was high in both groups; 86.7% in the CSF-aspiration group and 93.3% in the no-CSF-aspiration group. Although the mean time to reach the maximum level of anesthesia and to reach T10 level in the CSF-aspiration group is longer than the non-CSF-aspiration group, but this differences were not significant. Hypothetically, it can cause insufficient diffusion and decrease the level of spinal anesthesia or delay the onset of block (26, 32). Cerebrospinal fluid volume seems to be a key determinant of sensory block extent and motor block duration in SPA (33, 34). Given that lumbosacral CSF volume varies considerably between individuals (35), and regional anesthetic is diluted in the CSF before uptake into nerve roots and spinal cord. So, acute reduction in the volume of CSF can be change the effect of SPA (27). To the best of our knowledge, no previous CSF aspiration study in conjunction with SPA has examined acute reduction of CSF volume >1 mL. Pitkänen et al. (26) found no significant difference regarding pin-prick assessed maximum level of sensory block between groups receiving isobaric bupivacaine 15 mg (3 mL) with or without aspiration of 3 mL CSF prior to SPA in 60 elderly (58–77 years) orthopedic or urological patients. Kokki et al. (36) evaluated the effect of 1–3 mL CSF aspiration, equal to the weight-adjusted volume of levobupivacaine SPA, in 186 children aged 10 months to 18 years. No significant difference was found regarding extent of sensory block or duration of motor block, and there were no failed SPAs in the aspiration group. A prospective study was conducted by Cherng et al. (25), to assessed the effect on SPA of the dilution of isobaric 0.5% bupivacaine with CSF. Their findings showed only statistical difference in the time to reach complete motor block, which was shorter in group without CSF aspiration as compared to groups with aspiration. While no differences in onset time and duration of sensory block was observed between group with and without CSF aspiration. In contrast, Jawan and Lee (32), showed that aspiration of 5 mL of CSF before SPA with isobaric bupivacaine (10 mg, 2 mL) led to significantly higher sensory block compared to 3-mL aspiration and no aspiration in 66 patients who undergoing urological procedures. Recently, Bjurström et al. (27) reported that acute reduction of CSF volume by 10 mL prior to SPA with hyperbaric bupivacaine 0.5% (3 mL) leads to a higher thoracic level of sensory block.

In present study, the failure rate of SPA was not significant between two groups of study. However, it was 13.3% in the CSF-aspiration group vs. 6.7% in the no-CSF-aspiration group. Differences in outcomes between groups may be related to CSF aspiration and re-injection, which removes the needle tip from the spinal membrane (dura) and places it in the epidural space. On the other hand, the success of SPA depends on the competence of the anesthetic provider and has proven its effectiveness in skilled hands (37). This block must be performed with great care and method to reach a success rate of almost 100%. SPA in skilled hands is safer, although several factors are thought to affect the SPA such as the presence of anatomical disorders such as kyphoscoliosis, sclerosis, and spinal stenosis following previous intrathecal surgery or chemotherapy obesity and decreased potency of anesthetic drug due to prolonged exposure to light (38–40). The complete failure of spinal anesthesia is generally managed by conversion to general anesthesia or repeating the spinal anesthesia procedure. As assumed that all pregnant patients have a high risk of aspiration and difficulty for intubation, therefore, conversion to general anesthesia is associated with a relatively higher risk of the general population (41).

To our best knowledge, this study was the first CSF aspiration study exploring the effect of CSF aspiration volume <1 ml after injection SPA on the extent of sensory and motor block. The main limitation of the present study is the small sample size. So, further high-quality research with larger sample size is needed to strengthen the evidence base.

## Conclusion

The data indicate that the aspiration of 0.2 ml of CSF after injection of spinal anesthesia with hyperbaric 0.5% bupivacaine does not seem to affect the spread, duration, or outcome of SPA in cesarean section.

## Data Availability Statement

The original contributions presented in the study are included in the article/supplementary material, further inquiries can be directed to the corresponding author.

## Ethics Statement

The studies involving human participants were reviewed and approved by the Ethics Committees of Hamadan University of Medical Sciences (IR.UMSHA.REC.1399.630). The patients/participants provided their written informed consent to participate in this study.

## Author Contributions

FR-B, NM, and FE-A: study concept and design, and critical revision of the manuscript for important intellectual content. FR-B, NM, and ZM: analysis and interpretation of data. ZM: acquisition of data and drafting of the manuscript. ZM and FA: statistical analysis. All authors contributed to the article and approved the submitted version.

## Conflict of Interest

The authors declare that the research was conducted in the absence of any commercial or financial relationships that could be construed as a potential conflict of interest.

## Publisher’s Note

All claims expressed in this article are solely those of the authors and do not necessarily represent those of their affiliated organizations, or those of the publisher, the editors and the reviewers. Any product that may be evaluated in this article, or claim that may be made by its manufacturer, is not guaranteed or endorsed by the publisher.
